# Editorial Comment: Urologic surgery laparoscopic access: vascular complications

**DOI:** 10.1590/S1677-5538.IBJU.2015.0369.2

**Published:** 2017

**Authors:** Jose Jaime Correa

**Affiliations:** 1 Department of Urologic Oncology, Hospital Pablo Tobon Uribe, Medellin, Colombia

In the video by Branco et al. (1) two vascular injuries are shown. The video is very illustrative on how injuries are recognized and more important the way they are repaired. A quick intraoperative diagnosis of the injury and an appropiate management was performed by the surgeons.

Altough uncommon, an important percentage of injuries in laparoscopic procedures occur during the abdominal access using needles or trocars. In a recent Cochrane Systematic Review, no difference was found between direct trocar over Veress needle entry in terms of injuries (2). Complications can be minimized, but they can never be avoided. We have to know how to solve them when present. And as the authors mention in the abstract, you should never hesitate on converting to open surgery when necessary.

Jose Jaime Correa, MD Department of Urologic Oncology Hospital Pablo Tobon Uribe Medellin, Colombia E-mail: jocorreao@uces.edu.co
